# Influence of Flow Field on the Imaging Quality of Star Sensors for Hypersonic Vehicles in near Space

**DOI:** 10.3390/s25144341

**Published:** 2025-07-11

**Authors:** Siyao Wu, Ting Sun, Fei Xing, Haonan Liu, Kang Yang, Jiahui Song, Shijie Yu, Lianqing Zhu

**Affiliations:** 1 School of Instrument Science and Opto-Electronics Engineering, Beijing Information Science and Technology University, Beijing 100192, China; 2023030025@bistu.edu.cn (S.W.); 2023020357@bistu.edu.cn (H.L.); 2021020191@bistu.edu.cn (K.Y.); 2022020333@bistu.edu.cn (J.S.); yushijie@bistu.edu.cn (S.Y.); zhulianqing@bistu.edu.cn (L.Z.); 2 Laboratory of Intelligent Microsystems, Beijing Information Science and Technology University, Beijing 100192, China; 3 Department of Precision Instrument, Tsinghua University, Beijing 100084, China; xingfei@mail.tsinghua.edu.cn

**Keywords:** star sensor, aero-optical effect, ray tracing, star point offsets, image quality

## Abstract

When hypersonic vehicles fly in near space, the flow field near the optical window leads to light displacement, jitter, blurring, and energy attenuation of the star sensor. This ultimately affects the imaging quality and navigation accuracy. In order to investigate the impact of aerodynamic optical effects on imaging, the fourth-order Runge–Kutta and the fourth-order Adams–Bashforth–Moulton (ABM) predictor-corrector methods are used for ray tracing on the density data. A comparative analysis of the imaging quality results from the two methods reveals their respective strengths and limitations. The influence of the optical system is included in the image quality calculations to make the results more representative of real data. The effects of altitude, velocity, and angle of attack on the imaging quality are explored when the optical window is located at the tail of the vehicle. The results show that altitude significantly affects imaging results, and higher altitudes reduce the impact of the flow field on imaging quality. When the optical window is located at the tail of the vehicle, the relationship between velocity and offset is no longer simply linear. This research provides theoretical support for analyzing the imaging quality and navigation accuracy of a star sensor when a vehicle is flying at hypersonic speeds in near space.

## 1. Introduction

The near space refers to the airspace located 20 to 100 km above the Earth’s surface. It extends beyond the troposphere and covers the lower boundaries of the stratosphere, mesosphere, and thermosphere. In the integrated operations of aerospace and aviation in the field of military affairs, the near space plays a significant strategic role [1]. Hypersonic vehicles are representative of the high-speed platforms in near space that have developed rapidly in recent years. Trans-atmospheric reusable aircrafts and hypersonic unmanned aerial vehicles generally fly at hypersonic speeds (Mach numbers greater than 5) in near space. They typically possess advantages such as high flight speed, strong penetration capability, and good concealment performance [2]. Hypersonic vehicles in near space have great military and civilian value.

Star sensors offer high detection accuracy, low power consumption, and robust resistance to interference. They provide both position and attitude information for spacecrafts. However, when the vehicles fly at hypersonic speeds in near space, star sensors face new challenges regarding image quality. The aerodynamic conditions during hypersonic flight of these aircraft result in complex variations in fluid structure and temperature around optical windows [3]. This leads to non-uniform gradient states of fluid and optical window refractive indices. Images obtained through a time-varying refractive index field exhibit phenomena such as blurring, jitter, and energy attenuation, collectively referred to as aerodynamic optical transmission effects [4,5].

Extensive research has been conducted by numerous scholars on aerodynamic optical effects from different perspectives. In 2003, Yin provided a detailed introduction to theoretical and computational methods related to aerodynamic optics, as well as the aerodynamic optical effects and relevant correction methods [6]. Subsequently, in 2015, Boeing employed the two-dimensional Navier–Stokes equations to conduct an aerodynamic optical analysis of the flow field around concave windows [7]. In 2015, Chew L. and Christiansen W. detailed the variations in the time-averaged Strehl Ratio with the development of the boundary layer [8].

In terms of numerical calculations, the ray tracing theory has made significant progress in recent years, giving rise to various theories and methods. The ray equation has analytical solutions only under specific refractive index distributions. In irregular and non-uniform aerodynamic refractive index fields, numerical methods such as Euler’s method, the Taylor expansion method, and the Runge–Kutta method are generally employed. Among them, the third-order and fourth-order Runge–Kutta methods demonstrate higher accuracy and have become widely used in ray tracing. In 2019, Xiong et al. from the National University of Defense Technology proposed three fourth-order accuracy methods for ray tracing and compared their accuracy and speed with the results of spiral ray analytical solutions [9]. In 2021, Liu et al. proposed a new flow field ray tracing algorithm based on the three-dimensional cellular automaton theory and geometric optics theory [10]. However, the respective advantages and disadvantages of these methods have not been provided for tracking under hypersonic flow fields in near space. Moreover, these aerodynamic optical offset calculations are based on geometric optical models, assuming that light propagates along a straight line and ignoring diffraction effects. In the flow fields with low disturbance, the density gradient is small, and the PSF broadening caused by diffraction effects may dominate the offset. Using only geometric optical models would underestimate the actual offset. Therefore, we have supplemented the calculation formula for imaging quality based on the characteristics of the flow field in the hypersonic wake in near space. This method can provide a diffraction correction process for vehicles in the hypersonic state in near space.

In 2021, Yang et al. established a photon transport model in turbulent flow and designed a micro-mechanism-based simulation method for aero-optical effects, comparing optical distortion parameters based on photonics with physical quantities of traditional aero-optical effects and validating the effectiveness of microscopic analysis on a macroscopic scale [11]. In 2022, Liao et al. quantitatively assessed the performance of SWIR star sensors under aerodynamic conditions, focusing on mid-flight speeds (≤8 Ma). However, they did not explore the conditions of higher speeds, various velocities, and altitudes [12]. In 2024, Patel et al. performed a three-dimensional numerical simulation of a boundary layer and a shear layer flow and performed a spectral proper orthogonal decomposition of the optical path difference to ascertain the source of the aero-optical distortions [13]. It can be seen that previous studies have discussed the effect of the flow field from various perspectives, while we analyzed the specific process of the effect of the flow field on the imaging quality from the perspective of the density field.

The actual working condition involves the situation that the optical window is located at the tail of an aircraft in the vicinity of hypersonic speeds. However, previous simulation studies have mostly focused on the aircraft’s head and are limited to lower altitudes and high-speed flight conditions. Those studies lack the effects of the flow field around the tail of the aircraft under near-space hypersonic flight conditions.

In this paper, we compared two commonly used ray tracing methods in terms of their accuracy and computational efficiency under various step sizes and numbers of rays traced, and the accuracy and elapsed time of these two methods were analyzed. The result demonstrates the reliability of ray tracing results and facilitates the selection of appropriate methods and parameters for subsequent calculations in this study. Moreover, simulations under the near-space hypersonic flight conditions were conducted to investigate the flow field characteristics at different altitudes, velocities, and angles of attack. The influence of different flight conditions on the imaging quality of star sensors was analyzed and compared, providing theoretical support for the research on the navigation accuracy of star sensors when they are located at the tail of hypersonic aircraft in near space.

## 2. Ray Tracing Methods

**Figure 1:**
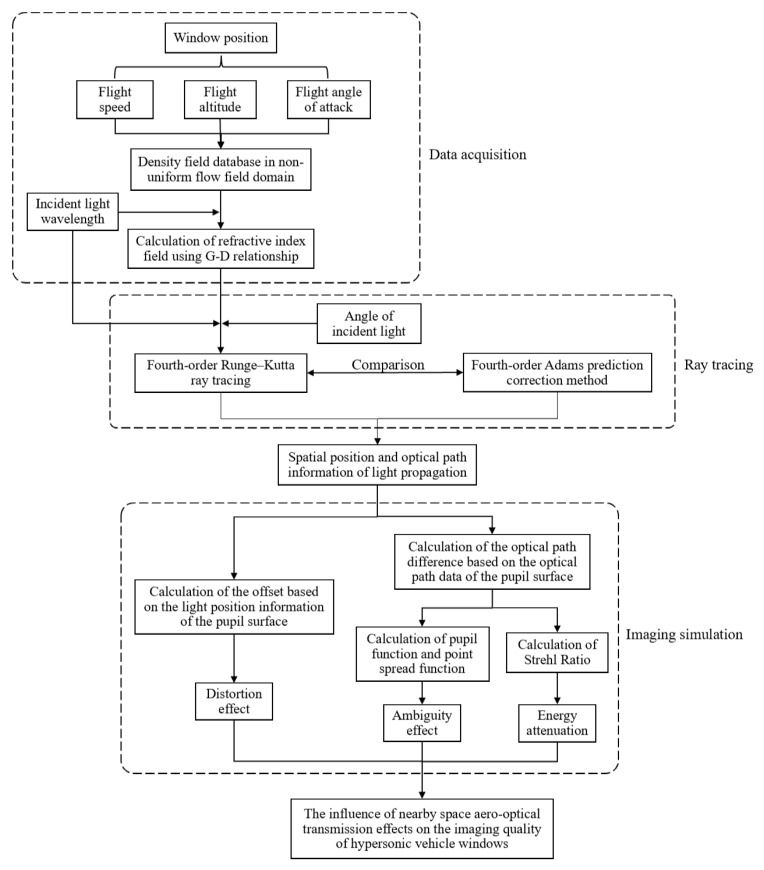
The study framework diagram.

The research on aerodynamic optical effects can be divided into three main parts: Firstly, using Fluent software, the flight environment is computationally analyzed to obtain the distribution of the density field around the aircraft under specific flight conditions. Secondly, based on the simulated density data, the ray-tracing method is employed to calculate the propagation path of light through the non-uniform flow field near the window. Finally, the imaging quality is evaluated by describing it in terms of the optical transfer function and Strehl Ratio. The research framework of this study is illustrated in Figure 1.

After inputting specific flight parameters, the density field near the optical window of the aircraft is obtained through an aerodynamic optical flow simulation to facilitate subsequent optical calculations. In this paper, the computational fluid dynamics software Fluent 2022R2 is adopted for the simulation of the flow field. The Geometry and Mesh modules within Fluent are utilized for modeling and mesh generation. In order to obtain better computational results, the grids near the window are densified during the grid generation phase, and the total number of grids is 1.57 million. The Spalart–Allmaras model is employed for iterative calculations based on the flow design of aerospace vehicles to obtain a database of the density field. The Spalart–Allmaras turbulence model was selected for its optimal balance between accuracy and computational efficiency in modeling the hypersonic boundary layer flow characteristics of near-space flight conditions. This model is particularly well-suited for wall-bounded flows and has been extensively validated for similar aero-optical applications [13,14]. Compared to alternative models, it provides robust performance in capturing the essential physics of shock–boundary layer interactions while maintaining computational tractability for our parametric study across multiple flight conditions.

The three-dimensional refractive index field can be obtained by calculating the density field database obtained through simulation using Fluent using the Lorentz–Lorenz equation, which can be expressed as [15]

(1) $$\left(\frac{n^{2}-1}{n^{2}+2}\right)\frac{1}{\rho }=\frac{2}{3}K_{GD}$$

where ρ is the refractive index, n is density, and $K_{GD}$ is the Gladstone–Dale coefficient, which usually takes the value in the standard state. It can be approximated as

(2) $$K_{GD}=2.23\times 10^{-4}\left(1+\frac{7.52\times 10^{-3}}{\lambda^{2}}\right)$$

Therefore, by utilizing the three-dimensional density field and wavelength, the refractive index field can be computed.

According to Fermat’s principle, light always propagates along the path of minimum optical path length. Therefore, when light propagates through a medium with continuous refractive index variations, the essential equation describing the propagation of light can be expressed as follows:

(3) $$\left\{\begin{array}{l} \frac{dr}{dp}=T\\ \frac{dT}{dp}=n\nabla n \end{array}\right.$$

where $p=\int \frac{ds}{n}$ and $T=\frac{dr}{dp}$ are variables, r is the position vector of a point along the light propagation trajectory, ds is a small step length along the light propagation trajectory, n is the refractive index value corresponding to the point on the light propagation trajectory, and ∇n is the refractive index value gradient corresponding to the point on the light propagation trajectory.

The light equation in the form of a second-order differential equation can be expressed in the form of a system of first-order differential equations:

(4) $$\begin{array}{l} \left\{\begin{array}{l} \frac{dr}{dp}=r\prime =T\\ r\prime (x_{0},y_{0},z_{0})=T_{0}=n_{0}\cdot k_{0} \end{array}\right.\\ \left\{\begin{array}{l} \frac{dT}{dp}=T\prime =n\nabla n\\ T\prime (x_{0},y_{0},z_{0})=K_{0}=n_{0}\nabla n_{0} \end{array}\right. \end{array}$$

The essence of the ray tracing method lies in the application of geometrical optics, where the trajectory of light rays in non-uniform media is traced based on the laws of refraction and the propagation of light. It enables the calculation of the position of the target ray on the image plane after passing through the flow field from the far-field region. Consequently, the influence of the flow field on optical imaging can be analyzed and evaluated [8].

The ray equation only has an analytical solution under specific refractive index distributions. In irregular and non-uniform aero-optical refractive index fields, numerical methods are generally required for a solution. Therefore, two ray tracing methods were selected for calculation. A comparison of the results of the two methods indirectly verifies the reliability of the results through the consistency of their outcomes.

### 2.1. The Fourth-Order Runge–Kutta Method

The light equation can be solved by using the fourth-order Runge–Kutta method. The system output and coefficients resulting from the solution using the fourth-order Runge–Kutta method are expressed as [16]

(5) $$\begin{array}{l} \left\{\begin{array}{l} r_{i+1}=r_{i}+\frac{h}{6}(K_{1}+2K_{2}+2K_{3}+K_{4})\\ K_{1}=T_{i}\\ K_{2}=T_{i}+\frac{h}{2}L_{1}\\ K_{3}=T_{i}+\frac{h}{2}L_{2}\\ K_{4}=T_{i}+hL_{3} \end{array}\right.\\ \left\{\begin{array}{l} T_{i+1}=T_{i}+\frac{h}{6}(L_{1}+2L_{2}+2L_{3}+L_{4})\\ L_{1}=n(r_{i})\nabla n(r_{i})\left(at\;r_{i}\right)\\ L_{2}=n(ra_{i})\nabla n(ra_{i})\left(at\;ra_{i}=r_{i}+\frac{h}{2}K_{1}\right)\\ L_{3}=n(rb_{i})\nabla n(rb_{i})\left(at\;rb_{i}=r_{i}+\frac{h}{2}K_{2}\right)\\ L_{4}=n(rc_{i})\nabla n(rc_{i})\left(at\;rc_{i}=r_{i}+hK_{3}\right) \end{array}\right. \end{array}$$

By employing the fourth-order Runge–Kutta tracing procedure, the current coordinates of the tracing point, $r_{i}$, and the coordinates of the next tracing point, $r_{i+1}$, can be obtained. The refractive index at each discrete point can be determined based on the coordinates, $r_{i}$, and the geometric path length from the current point to the next tracing point, $l_{i}$.

The optical path length (*OPL*) of a light ray is defined as the product of the distance traveled by the light ray and the refractive index of the medium through which the light ray passes. Let us consider that the transmission path of the *i*-th light ray through the flow field is denoted by $l_{i}$. Along this path, the $OPL_{i}$ of the light ray can be computed as follows:

(6) $$OPL_{i}=\sum_{i-1}^{n}n_{i}l_{i}$$

**Figure 2:**
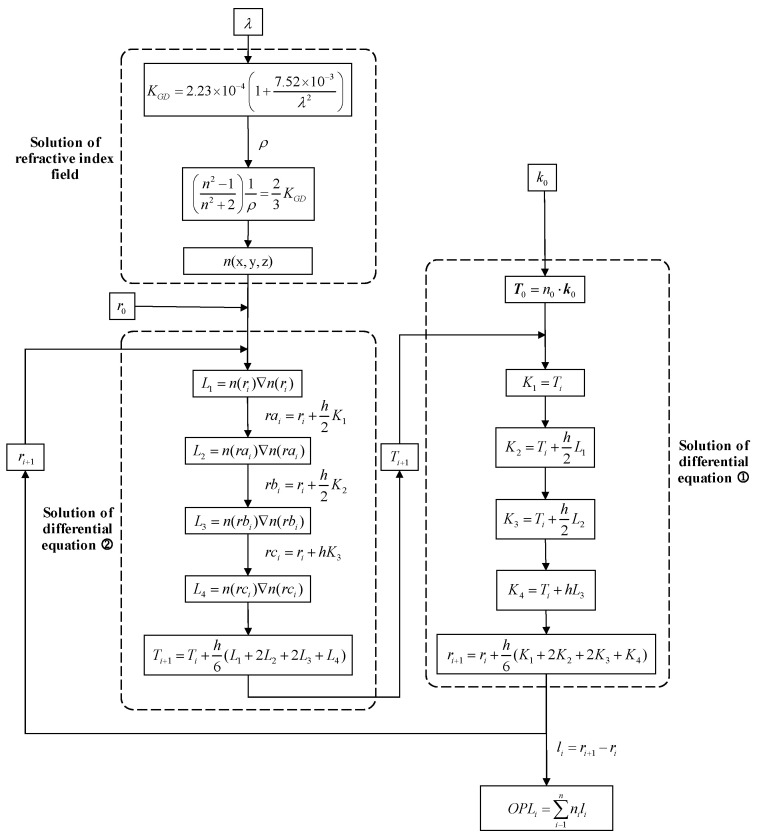
The solving process of the fourth-order Runge-Kutta method.

The overall solution process of the ray equations using the fourth-order Runge–Kutta method is illustrated in Figure 2.

### 2.2. Fourth-Order Adams–Bashforth–Moulton (ABM) Predictor-Corrector Method

The Adams–Bashforth method is a numerical solver for first-order ordinary differential equations. By applying the Adams–Bashforth method, the system of first-order ordinary differential equations can be determined. Therefore, (4) can be solved [9]:

Predict:

(7) $$\left\{\begin{array}{l} {\bar{r}}_{i+1}=r_{i}+\frac{h}{24}(55K_{i}-59K_{i-1}+37K_{i-2}-9K_{i-3})\\ {\bar{T}}_{i+1}=T_{i}+\frac{h}{24}(55L_{i}-59L_{i-1}+37L_{i-2}-9L_{i-3}) \end{array}\right.$$

Correct:

(8) $$\left\{\begin{array}{l} r_{i+1}=r_{i}+\frac{h}{24}(9K_{i+1}+19K_{i}-5K_{i-1}+K_{i-2})\\ T_{i+1}=T_{i}+\frac{h}{24}(9L_{i+1}+19L_{i}-5L_{i-1}+L_{i-2}) \end{array}\right.$$

Due to the inability of the Adams predictor-corrector method to be self-starting, the preceding four steps employ the fourth-order Runge–Kutta method to obtain the required parameters. Additionally, both methods utilize a consistent step size throughout their computational procedures.

## 3. Calculation of Image Quality

The calculation of image quality involves the application of ray tracing to track the propagation path of light rays through a flow field. Integrating along the path allows us to determine the optical path difference (OPD) at the entrance pupil. By constructing the entrance pupil function from the OPD, we can apply physical optics methods to obtain the complex amplitude distribution of the wavefront after passing through the flow field. From the complex amplitude distribution on the image plane, various parameters can be calculated, including the point spread function, modulation transfer function, image shift, and Strehl Ratio.

### 3.1. Calculation of Image Quality Evaluation Function

After obtaining the optical path using ray tracing, the calculation of image quality can be performed. The OPD is defined as the difference between the optical paths of the discussed light ray and the reference light ray, typically the chief ray in a bundle of rays. The difference in optical paths between each ray and the chief ray is expressed as [17]

(9) $$w\left(x,y\right)=\frac{2\pi }{\lambda }OPD=\frac{2\pi }{\lambda }\left(OPL_{i}-OPL_{0}\right)$$

By calculating the optical path difference of each ray on the pupil, we obtain the wavefront aberration of the entire exit pupil, which can be expressed as

(10) $$A\left(x,y\right)=\alpha \left(x,y\right)e^{j\frac{2\pi }{\lambda }w\left(x,y\right)}$$

According to the Huygens principle, the Fourier transform of the pupil function yields the amplitude distribution of the image plane, which can be expressed as

(11) $$U\left(x\prime ,y\prime \right)=\iint A\left(x,y\right)e^{j\frac{2\pi }{\lambda f\prime }\left(xx\prime +yy\prime \right)}dxdy$$

As the intensity is proportional to the square of the amplitude, the point spread function can be represented as [17]

(12) $$PSF\left(x\prime ,y\prime \right)={\left|U\left(x\prime ,y\prime \right)\right|}^{2}$$

The PSF describes how star light is spatially distributed after passing through the disturbed optical path, determining the fundamental resolution limit and blurring characteristics.

By Fourier transforming the point spread function, we can obtain the optical transfer function of the laminar flow field, which can be expressed as

(13) $$OTF\left(f_{x},f_{y}\right)=\iint PSF\left(x\prime ,y\prime \right)e^{-j2\pi \left(f_{x\prime }x\prime +f_{y\prime }y\prime \right)}dx\prime dy\prime$$

The modulation transfer function (MTF) is a measure of the optical transfer function, which can be expressed as

(14) $$MTF=\sqrt{OTF\cdot OTF^{*}}$$

The MTF measures the ability of the system to maintain image contrast across different spatial frequencies.

The laminar flow not only generates blurring but also introduces displacement. The point spread function (PSF) distribution, obtained by passing the target light through the flow field and imaging it onto the focal plane of an optical imaging system, reaches its maximum value at coordinates $\left(x_{\max}^{\prime },y_{\max}^{\prime }\right)$. These coordinates correspond to the image shift induced by laminar flow. The image displacement characterizes the global shift of the image plane due to refractive index gradients, directly affecting the absolute position measurement accuracy.

The Strehl Ratio (SR) is a measure of the ratio of the maximum intensity in the diffracted image with aberrations to that without aberrations. The Strehl Ratio evaluates the overall wavefront quality through peak intensity reduction. It is used to characterize the impact of near-field distortions, caused by the non-uniform flow field, on the far-field propagation characteristics of a beam. The Strehl Ratio is a parameter that represents the intensity decay of an image. Under the assumption of a large aperture approximation, the root mean square of the optical path difference $OPD_{rms}$ and the wavelength λ form a function that measures the quality of the intensity distribution of an optical system. The calculation formula is as follows [18]:

(15) $$SR=\exp\left[-{\left(\frac{2\pi \cdot OPD_{rms}}{\lambda }\right)}^{2}\right]$$

The magnitude of the optical transmission effects in high-speed flow fields is described by the resulting image displacement, image blur, and image jitter. Image displacement is caused by the density, density gradient, and flow field structure, which is equivalent to the eccentric lens effect, measured by the angle relative to the target sightline or the distance of the target image point relative to when there is no flow field. Image blur is generated by laminar flow and high-frequency turbulent flow, measured by the PSF, MTF, or Strehl Ratio. Image jitter manifests as motion of the imaging position on the detector plane, measured by the range of imaging position jitter, probability distribution of jitter, and jitter frequency. In this paper, the image displacement, PSF, MTF, and Strehl Ratio are selected as imaging indicators, which can describe the imaging quality from different aspects, and the conclusions drawn from different indicators should be consistent.

### 3.2. Analysis of Overall Imaging Quality Conducted Through Computational Method

**Figure 3:**
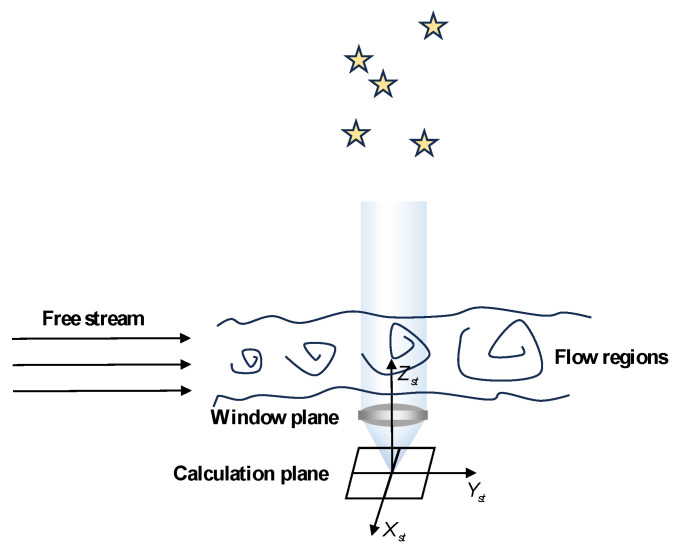
Schematic diagram illustrating the overall ray tracing path of incident light perpendicular to the window plane.

The overall ray tracing process is divided into two parts: ray tracing in the non-uniform flow field and ray tracing within the optical system. Ray tracing in the non-uniform flow field starts from the far-field region and traces to the window plane, which corresponds to the entrance pupil of the optical system. Ray tracing within the optical system starts from the window plane and traces to the calculation plane, representing the image plane. When the rays are incident vertically on the window plane, the overall ray tracing path is shown in Figure 3.

By summing the optical path differences before and after the entrance pupil of the optical system, the total optical path difference from the starting tracing position in the far-field and stable flow region to the image plane is obtained. Then, various image quality evaluation functions can be calculated.

Before the light enters the entrance pupil of the optical system, the trajectory of the light in the flow field region is traced. If the system behind is considered as an ideal optical system, the optical path difference at the exit pupil, which is obtained by tracing the light trajectory before entering the entrance pupil, is equal to the optical path difference when reaching the entrance pupil because an ideal optical system has no optical path difference. However, to make the results closer to the actual situation, we no longer consider ideal optical systems. We considered the impact of real optical systems on imaging results and provide an explanation in Section 3.2.

### 3.3. Calculation of Imaging Displacement

Due to the fact that both the actual operating conditions and the simulated conditions involve the tail section of a hypersonic aircraft in near space, the resulting image displacement is small. The image displacement obtained through ray tracing approaches zero. Therefore, in order to capture the aerodynamic optical transmission effects on the tail section of a hypersonic aircraft in near space, it is necessary to improve the precision of the results.

The point spread function obtained from ray tracing calculations with a quantity of 641 × 641 rays form a matrix of the same size. However, the precision of the point spread function obtained with the current quantity of rays is insufficient. Increasing the number of rays for tracing will significantly increase the overall computational time of the program. Therefore, we initially considered using interpolation methods to obtain higher precision point spread functions. Common interpolation methods include nearest-neighbor interpolation, linear interpolation, and cubic spline interpolation. Cubic spline interpolation constructs a cubic polynomial within each interval, satisfying the interpolation conditions while ensuring continuous first and second derivatives at each node. This interpolation method achieves higher precision than both the nearest-neighbor and linear interpolation methods. In many practical applications, cubic spline functions outperform other higher-order spline functions in terms of interpolation accuracy [19].

However, through our actual calculations, we found that interpolation methods cannot effectively resolve the computational challenges posed by extremely small imaging displacements. Moreover, the interpolation approach may introduce computational artifacts or uncertainties into the final results. Therefore, we adopted the method of calculating the centroid of the PSF to determine the imaging displacement.

In addition, traditional methods for calculating light offset are suitable for imaging against an ideal optical system, but we need to focus more on the actual flow field situation. Moreover, the refractive index changes are small in some of the adjacent spatial scales, which makes the use of this calculation less refined. Therefore, we analyze the light offset situation in more detail.

The fluctuating nature of light allows the diffraction of an ideal point source after passing through an ideal optical system, resulting in a diffuse spot on the image plane. For diffraction-limited optical systems, the point spread function (PSF) behaves as an Airy spot. The Airy spot is the diffraction-limited response of a point source in an ideal optical system (no aberration, circular aperture), and its light intensity distribution is described by the Bessel function:

(16) $$I(r)={\left[\frac{2J_{1}\left(kr\right)}{kr}\right]}^{2}$$

where $k=\frac{\pi D}{\lambda f}$, *J*_1_ is the Bessel function of the first type, *D* is the diameter of the incident pupil of the optical system, *λ* is the wavelength of the light, and *f* is the focal length of the system. The grayscale distribution of the ideal light source on the image plane is calculated by Airy points:

(17) $$PSF_{Airy}\left(x\prime ,y\prime \right)={\left[\frac{2J_{1}\left(\frac{\pi D}{\lambda f}\sqrt{x\prime^{2}+y\prime^{2}}\right)}{\frac{\pi D}{\lambda f}\sqrt{x\prime^{2}+y\prime^{2}}}\right]}^{2}$$

The grayscale distribution of the ideal light source on the image plane, calculated by the ideal light source through the Airy spot, is approximated as a convolution kernel K:

(18) $$K\left(x\prime ,y\prime \right)=PSF_{Airy}\left(x\prime ,y\prime \right)$$

Convolutional computation of the PSF with K is performed to realize the diffusion process for imaging:

(19) $$PSF_{\mathrm{conv}}\left(x\prime ,y\prime \right)=PSF\left(x\prime ,y\prime \right)\cdot K\left(x\prime ,y\prime \right)$$

Finally, we compute the center of mass of the convolved PSF to obtain the imaging offset:

(20) $$\left\{\begin{array}{l} \Delta_{x}=\frac{\sum_{i,j}x_{i,j}\cdot PSF_{\mathrm{conv}}\left(x_{i,j}\prime ,y_{i,j}\prime \right)}{\sum_{i,j}PSF_{\mathrm{conv}}\left(x_{i,j}\prime ,y_{i,j}\prime \right)}\\ \Delta_{y}=\frac{\sum_{i,j}y_{i,j}\cdot PSF_{\mathrm{conv}}\left(x_{i,j}\prime ,y_{i,j}\prime \right)}{\sum_{i,j}PSF_{\mathrm{conv}}\left(x_{i,j}\prime ,y_{i,j}\prime \right)} \end{array}\right.$$

## 4. Comparison of Two Ray Tracing Methods

In order to verify the feasibility and effectiveness of the two algorithms and obtain reliable experimental results for subsequent experiments, this study compares two commonly used algorithms from the perspective of quantitative indicators. Specifically, for the density field of a specific operating condition (Mach number 15, −4.7° angle of attack, 60 km altitude), the fourth-order Runge–Kutta method and the fourth-order Adams predictor-corrector method are employed to trace the flow field at the tail of the aircraft using ray tracing. The tracing results are then compared under different step sizes of traced rays.

In order to obtain accurate coordinate data and consider the appropriate tracking time and tracking accuracy, the subsequent calculation will use 321 × 321 tracking rays. To provide a clearer comparison of tracking times, a quantitative approach is employed for comparing tracking times. A tracking step size of 6 mm is selected, and the tracking time using the fourth-order Runge–Kutta method serves as the quantitative standard. The simulation was performed using Fluent2022 and Matlab R2023b with the CPU at 2.5 GHz. The computing time of the quantitative standard condition is 95 min.

By means of the above experimental results, the results obtained by the two ray tracing methods can be validated. Since an analytical solution cannot be directly obtained for the ray equations, we consider the high-precision results obtained with a larger number of traced rays and smaller step sizes as the ground truth. From Table 1, it can be observed that under high-precision conditions, the results obtained by the tracing methods are consistent and can be regarded as accurate, whereas under low-precision conditions, there will be some deviations in the results. Under the same conditions of traced ray count and step size, the fourth-order Adams predictor-corrector method exhibits shorter tracing time but lower precision compared to the fourth-order Runge–Kutta method. When high-precision tracing results are required, the fourth-order Runge–Kutta method can be employed, whereas when dealing with a large amount of data and when the non-uniform flow field has a significant impact on the image quality, the fourth-order Adams predictor-corrector method can be utilized. Since this study focuses on the flow field near the rear window of a hypersonic vehicle in near space, where the impact of the non-uniform flow field on image quality is minimal and high-precision tracing results are necessary, the fourth-order Runge–Kutta method is chosen for ray tracing. In order to ensure result accuracy, larger step sizes and an appropriate number of traced rays should be selected as much as possible. Consequently, the subsequent ray tracing process will adopt a 6 mm step size and employ the fourth-order Runge–Kutta method with a ray count of 321 × 321 for the tracing calculations.

## 5. Results and Discussion

The flight conditions, as well as the location and shape of the flight window, can influence the characteristics of the flow field surrounding the aircraft, thereby affecting the transmission path of light and the quality of imaging. In order to comprehensively analyze the influence of the aerodynamic flow field on the transmission characteristics of the flow field at the rear of the aircraft during near-space hypersonic flight, this paper conducts simulation calculations on typical operating conditions. By calculating the image quality under different operating conditions, the relationship between the flow field conditions and imaging quality can be quantitatively described. Moreover, the resulting data and trends can provide support for subsequent star map reconstruction research.

The different groups of operating conditions for Fluent simulation are presented in Table 2, where S denotes simulation, representing the simulated operating conditions.

**Figure 4:**
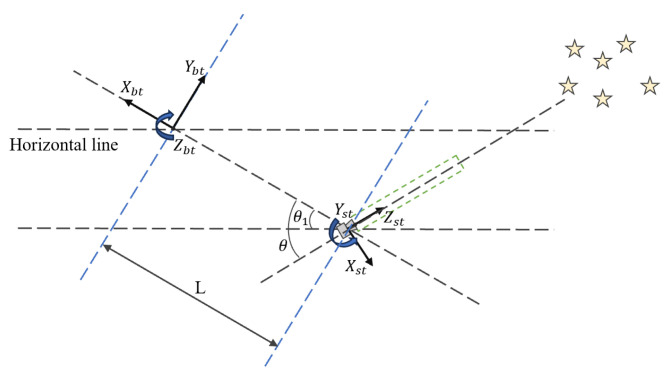
Schematic diagram of the mathematical coordinate system for aircraft and star sensors.

The density field data of the wake flow is simulated through the mathematical coordinate system $o-x_{st}y_{st}z_{st}$ of the star tracker, as shown in Figure 4. After obtaining the actual density field database, it is necessary to convert data from the aircraft coordinate system $o-x_{bt}y_{bt}z_{bt}$ to the star tracker casing coordinate system and then to the mathematical coordinate system of the star tracker.

### 5.1. Influence of Velocity on Image Quality

**Figure 5:**
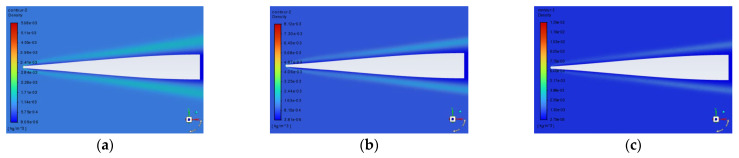
Typical density fields near the window at different flight speeds of (**a**) 8 Ma, (**b**) 12 Ma, and (**c**) 16 Ma.

Altitude affects atmospheric pressure and temperature, consequently impacting the density near the windows [20]. Therefore, when conducting simulations under different altitude conditions using Fluent, the temperature and pressure need to be configured. The distribution of flow characteristics, specifically the density field distribution, near the optical window of the spacecraft can be obtained through fluent simulation. The typical variation patterns of the density field distribution near the window at different speeds are shown in Figure 5.

By comparing density field plots at different flight speeds, it is observed that with the increase in flight speed, the size of the shock layer region decreases, while the maximum density value in the shock layer region increases. Therefore, the influence of flight speeds on the imaging quality cannot be obtained directly from the characteristics of the shock waves in the flow field regions but needs to be obtained by quantitative calculation.

**Figure 6:**
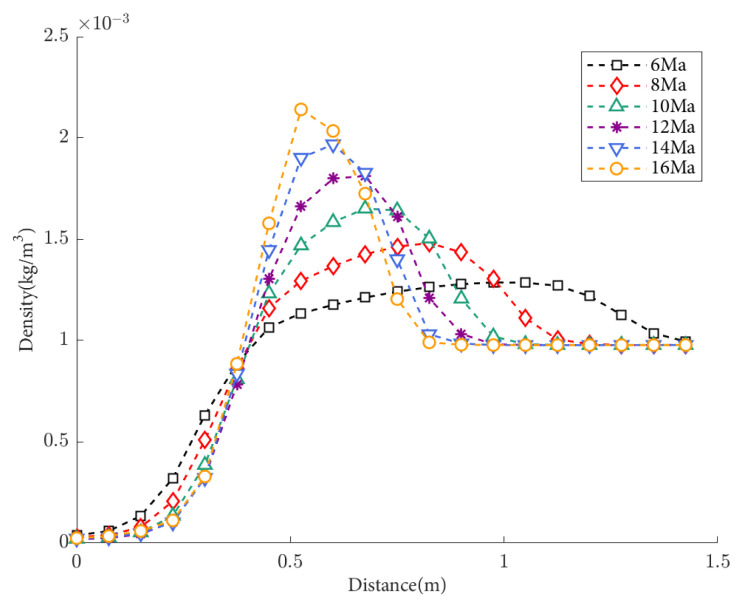
Density distribution of the flow field at different flight speeds with a flight altitude of 50 km and an attack angle of 0.

The density distribution of flow field density under condition S1 (50 km, 0° angle of attack) are shown in Figure 6. The abscissa represents the distance between the center-tracing ray of the window and the window plane. When the flight altitude remains constant, the changes in the flow field near the window become more complex with increasing flight speed, and the parameters such as density also vary in a more complex manner. In the far field of the flow field, the refractive index tends to stabilize. The greater the density, the greater the degree of light deflection. As the flight speed increases, the density peak becomes larger, but the distance of atmospheric disturbance decreases. Therefore, it is not possible to qualitatively determine the relationship between speed and the amount of light deflection through density distribution alone. Consequently, it is necessary to calculate the deflection using ray tracing and then determine the relationship between the two.

**Figure 7:**
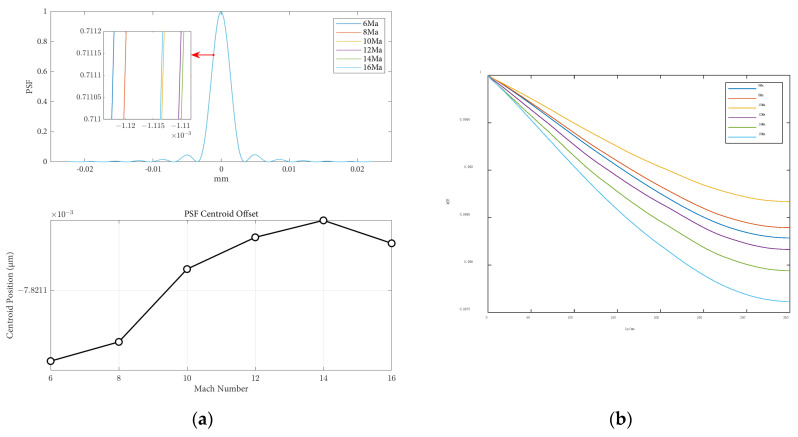
Optical evaluation functions at different flight speeds: (**a**) the PSF results and centroid offsets varied with flight speeds; (**b**) the MTF results varied with flight speeds.

By performing ray tracing and evaluating the image quality function, we obtain the effect of Mach number changes on the point spread function and modulation transfer function, and the light is incident vertically, as shown in Figure 7. It can be observed that under the same flight conditions and optical parameters in near space, the impact of flight speed on image quality is relatively minor. By examining the modulation transfer function charts, it can be seen that within the flight speed range of 6 to 10 Mach, as the flight speed increases, the MTF curve gradually rises, indicating a decrease in aerodynamic light transmission effects near the optical window. On the other hand, within the same speed range, as the flight speed increases, the MTF curve gradually declines, indicating an increase in aerodynamic light transmission effects near the optical window, leading to a decrease in image quality of the optical system.

Table 3 illustrates the effects of Mach number variation on image displacement and the Strehl Ratio when light enters vertically at different speeds. When the flight speed is in the range of 6 to 10 Mach, a faster flight speed results in a smaller impact of aerodynamic optical effects on optical imaging quality, leading to clearer imaging and a smaller point spread function. As the flight speed increases, the Strehl Ratio also increases, indicating a weakening of aerodynamic optical effects. However, when the flight speed is in the range of 10 to 16 Mach, a faster flight speed leads to a greater impact of aerodynamic effects on optical imaging quality, resulting in blurrier imaging and a larger PSF. In this case, a faster flight speed corresponds to a smaller Strehl Ratio, indicating an enhancement of aerodynamic optical effects. Overall, during hypersonic flight at an altitude of 50 km, the influence of flight speed on imaging offset is negligible, and the Strehl Ratio consistently approaches one, indicating good imaging quality.

Based on the calculations and analyses of various image quality evaluation indicators, namely the PSF curve, MTF curve, imaging offset, and Strehl Ratio, they consistently characterize image quality. These results also align with the findings of imaging effects obtained through a density analysis of the flow field, thereby providing indirect confirmation of the accuracy of the aerodynamic optical effect calculation model established in this study.

### 5.2. Influence of Altitude on Image Quality

**Figure 8:**
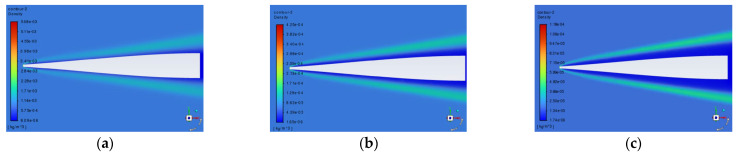
Typical density fields near the window at different flight altitudes of (**a**) 50 km, (**b**) 70 km, and (**c**) 90 km.

The typical variation patterns of the density field distribution near the window at different altitudes are shown in Figure 8. Through the comparison of density field maps at different flight altitudes, it was observed that as the flight altitude increases, the shock layer region decreases, the shock layer moves further away from the window plane, and the maximum density value decreases. This is because altitude affects atmospheric parameters such as pressure and temperature, which in turn affect the density near the window. The maximum density value is also influenced by atmospheric boundary conditions. By analyzing the density field at different flight altitudes, we can conclude that the effect on the imaging quality is greater at lower altitudes.

**Figure 9:**
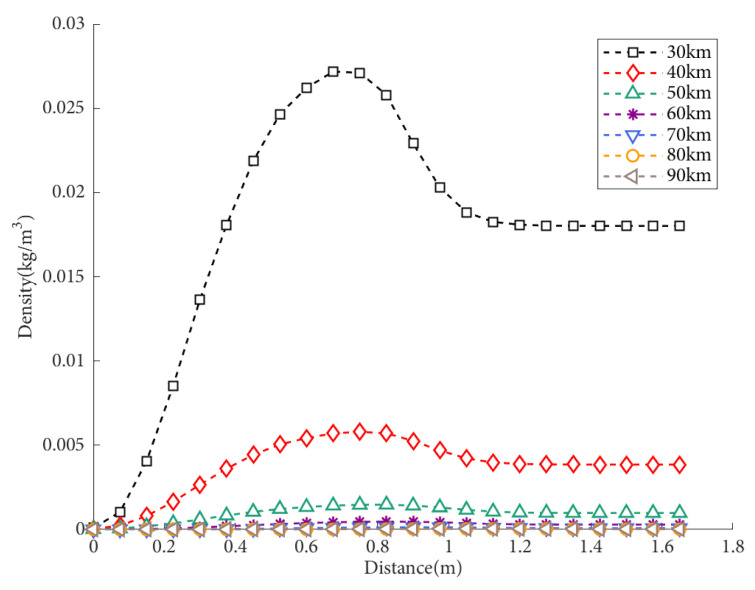
Density distribution of the flow field at different flight heights, with a flight speed of Mach 8 and an angle of attack of 0.

The density distribution of the flow field density under condition S2 (8 Mach, 0° angle of attack) is shown in Figure 9. As the flight altitude increases, the density peak decreases, the shock layer becomes closer to the aircraft wall, and the distance of atmospheric disturbance remains relatively constant. When the flight altitude is higher, the density near the window hardly changes, and the aerodynamic optical effects are minimal. As the density increases, the degree of light deflection increases. By analyzing the density distribution pattern, it is possible to qualitatively determine the relationship between altitude and the offset of light passing through the flow field. Specifically, as the altitude increases, the density peak in the flow field becomes smaller, the density variation decreases, the imaging offset decreases, the aerodynamic optical effects weaken, and the imaging quality improves.

**Figure 10:**
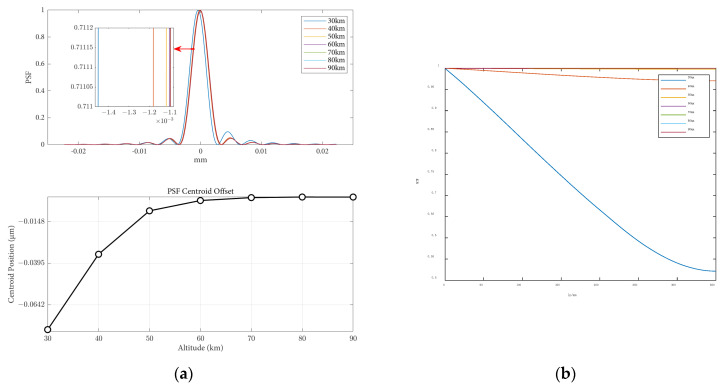
Optical evaluation functions at different flight heights: (**a**) the PSF results and centroid offsets varied with flight heights; (**b**) the MTF results varied with flight heights.

The influence of altitude variations on the point spread function and modulation transfer function, when the angle of attack is 0 and the speed is 8 Mach, is shown in Figure 10. From the figure, it can be observed that under the same flight conditions and optical parameters, the MTF curve gradually increases with increasing flight altitude. Within the range of 40 km altitude, the MTF value changes significantly with altitude. As the altitude continues to increase, the change in the MTF value becomes smaller. This is because with higher altitudes, the atmospheric density becomes thinner, and the aerodynamic optical transmission effects of the optical hood on imaging quality become weaker.

The influence of height variations on the imaging offset and Strehl Ratio is shown in Table 4. It can be observed that as the height increases, the impact of aerodynamic optical transmission effects on imaging offset decreases, resulting in smaller point spread functions. Additionally, with increasing flight height, the loss of light intensity decreases, leading to an increase in the Strehl Ratio. The influence of aerodynamic optical effects on image quality gradually diminishes. The pattern of Strehl Ratio results obtained with varying flight heights is similar to the results obtained using different methods by Guo, as reported in the existing literature [21], further confirming the correctness of the aerodynamic optical effects calculation model proposed in this paper. In close proximity to the target space, flight height has a significant impact on imaging offset, and the Strehl Ratio results all approach one, indicating good image quality. The results of the Strehl Ratio and the MTF obtained both suggest that as flight height increases, the aerodynamic optical transmission effects of the optical window attenuate, thereby validating the consistency of the image quality evaluation metrics for optical transmission once again.

### 5.3. Effect of Angle on Image Quality

**Figure 11:**
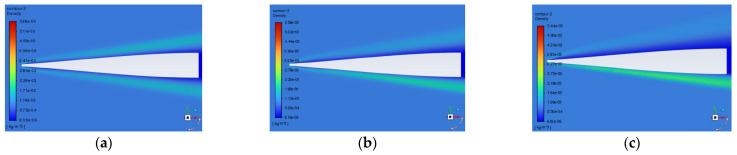
Typical density fields near the window at different angles of attack of (**a**) 0°, (**b**) 5°, and (**c**) 10°.

The typical variation patterns of the density field distribution near the window at different angles of attack are shown in Figure 11. By comparing the density fields at different angles of attack, it is observed that with the increase in the flight angle of attack, the region of shock waves gradually increases, but the density of the region of shock waves decreases. Therefore, the influence of the angle of attack on the imaging quality cannot be obtained directly from the characteristics of the shock waves in the flow field regions but needs to be obtained by quantitative calculation.

**Figure 12:**
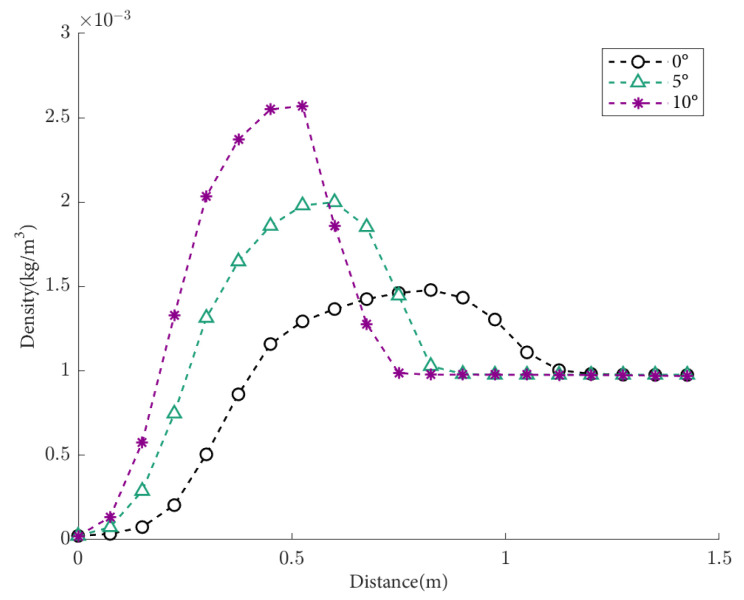
Density distribution of the flow field at different attack angles, with a height of 50 km and a flight speed of Mach 8.

The density distributions of the flow field density under condition S3 (8 Mach, 50 km) are shown in Figure 12. As the flight attack angle (positive angle of attack) increases, the density peak increases, the distance of atmospheric disturbance decreases, and the density change region of the flow field becomes closer to the aircraft’s surface. When the attack angle is 0°, the far-field refractive index region remains stable.

**Figure 13:**
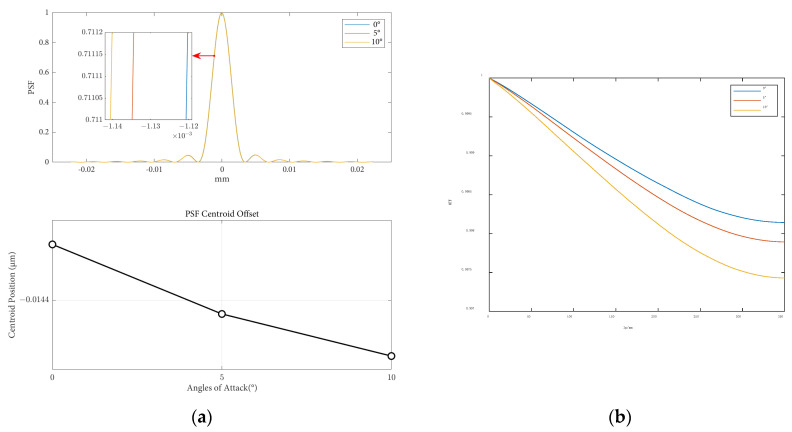
Optical evaluation functions at different attack angles: (**a**) the PSF results and centroid offsets varied with flight heights; (**b**) the MTF results varied with flight heights.

The influence of attack angle variation on the point spread function and modulation transfer function is shown in Figure 13 for a flight altitude of 50 km and a speed of 8 Ma.

The impact of the angle of attack variation on image displacement and the Ratio is shown in Table 5. As the angle of attack increases, the aerodynamic effects have a greater influence on optical image quality, resulting in a larger point spread function (PSF). Additionally, with an increase in the angle of attack, there is an increase in light intensity loss, a decrease in the Strehl Ratio, and an enhancement of aerodynamic optical effects.

## 6. Conclusions

This study focuses on the analysis of the tail conformal window of near-space hypersonic aircraft. Using the computational fluid dynamics software Fluent, the fluid flow distribution near the optical window under various flight conditions was simulated. Based on three-dimensional density field data, two ray tracing methods were compared. The ray tracing method suitable for the current simulation environment was selected, and the calculation method of image quality evaluation was proposed for the actual flight conditions of the aircraft. The imaging quality under different flight conditions was calculated, and corresponding optical evaluation functions and Strehl Ratios were provided. The following conclusions can be drawn:

(1) When high-precision tracking results are required, the fourth-order Runge–Kutta method can be used. When handling a large amount of data and the imaging quality is greatly affected by non-uniform flow fields, the fourth-order Adams predictor-corrector method can be utilized.
(2) Under near-space hypersonic flight conditions, the impact of flight altitude on imaging results is a more important research factor. As the altitude increases, the influence of aerodynamic optical effects decreases, resulting in reduced imaging displacement and an increase in the Strehl Ratio. Therefore, higher altitudes generally result in better imaging quality. In lower-altitude regions, greater attention needs to be paid to the impact of aerodynamic optical effects on image quality.
(3) Under near-space hypersonic flight conditions, flight velocity and angle of attack have a minimal influence on imaging results, indicating that imaging quality is not sensitive to changes in these factors. The relationship between velocity and offset is not linear when the optical window is facing towards the tail.

The method and the experimental results provide data and theoretical support for star sensor navigation accuracy analysis. Although this paper simulates the flow field near space at different altitudes, velocities, and angles of attack, there is a lack of research on the influence of window shape on imaging quality. Therefore, future studies could include simulations and analyses of different window shapes. Additionally, based on the data this work obtained, future research could focus on imaging offset compensation. We plan to conduct additional experiments using our methodology to obtain more comprehensive results. By employing neural network approaches, we aim to predict both image degradation and displacement patterns, thereby achieving effective compensation for navigation imaging errors in star sensors caused by flow field effects.

## Figures and Tables

**Table 1:** Tracing offset and time of vertical incidence for various methods and step sizes.

Tracing Method	Step Size (mm)	X-Direction Line Offset (μm)	Y-Direction Line Offset (μm)	Tracing Time
Fourth order Runge–Kutta method	3	2.83 × 10^−1^	7.10 × 10^−2^	1.91
	6	2.83 × 10^−1^	7.10 × 10^−2^	1
	8	2.83 × 10^−1^	6.90 × 10^−2^	0.82
Fourth-order ABM predictor-corrector method	3	2.83 × 10^−1^	7.13 × 10^−2^	0.94
	6	2.81 × 10^−1^	6.90 × 10^−2^	0.56

**Table 2:** Simulation flow field density database information.

Group of Operating Conditions	Mach Number	Altitude	Angle of Attack	Window Position
S1	6~16 Ma	50 km	0°	Tail
S2	8 Ma	20~90 km	0°	Tail
S3	8 Ma	50 km	0~10°	Tail

**Table 3:** Image displacement and Strehl Ratio at different speeds.

Speed (Ma)	X-Direction Line Offset (μm)	Y-Direction Line Offset (μm)	Strehl Ratio (%)
6	−2.45 × 10^−2^	4.23 × 10^−2^	99.81
8	−2.23 × 10^−2^	4.23 × 10^−2^	99.82
10	−1.56 × 10^−2^	4.01 × 10^−2^	99.85
12	−1.34 × 10^−2^	4.90 × 10^−2^	99.80
14	−1.11 × 10^−2^	5.12 × 10^−2^	99.77
16	−1.56 × 10^−2^	5.57 × 10^−2^	99.74

**Table 4:** Image displacement and Strehl Ratio at different heights.

Height (km)	X-Direction Line Offset (μm)	Y-Direction Line Offset (μm)	Strehl Ratio (%)
30	−2.41 × 10^−1^	4.96 × 10^−1^	51.00
40	−1.79 × 10^−2^	5.05 × 10^−2^	96.81
50	−3.88 × 10^−3^	1.32 × 10^−2^	99.76
60	−1.26 × 10^−3^	4.15 × 10^−3^	99.98
70	−2.83 × 10^−4^	1.44 × 10^−3^	99.997
80	−7.65 × 10^−5^	5.02 × 10^−4^	99.9997
90	−8.65 × 10^−5^	5.74 × 10^−4^	99.9996

**Table 5:** Image displacement and Strehl Ratio at different angles of attack.

Angles of Attack (°)	X-Direction Line Offset (μm)	Y-Direction Line Offset (μm)	Strehl Ratio (%)
0	−3.88 × 10^−3^	1.32 × 10^−2^	99.76
5	−7.22 × 10^−3^	1.03 × 10^−2^	99.77
10	−8.82 × 10^−3^	1.05 × 10^−2^	99.73

## Data Availability

The synthetic data supporting the results of this study are available from the corresponding author upon reasonable request.
